# CoNTe: A Core Network Temporal Blockchain for 5G

**DOI:** 10.3390/s20185281

**Published:** 2020-09-15

**Authors:** Steven Platt, Luis Sanabria-Russo, Miquel Oliver

**Affiliations:** 1 Department of Information and Communications Technologies, Universitat Pompeu Fabra, 08005 Barcelona, Spain; miquel.oliver@upf.edu; 2 Telecommunications Technological Centre of Catalonia (CTTC/CERCA), 08860 Castelldefels, Spain; luis.sanabria@cttc.es

**Keywords:** blockchain, 5G-specific blockchain, software-defined networks, network functions virtualization, distributed algorithms

## Abstract

Virtual Network Functions allow the effective separation between hardware and network functionality, a strong paradigm shift from previously tightly integrated monolithic, vendor, and technology dependent deployments. In this virtualized paradigm, all aspects of network operations can be made to deploy on demand, dynamically scale, as well as be shared and interworked in ways that mirror behaviors of general cloud computing. To date, although seeing rising demand, distributed ledger technology remains largely incompatible in such elastic deployments, by its nature as functioning as an immutable record store. This work focuses on the structural incompatibility of current blockchain designs and proposes a novel, temporal blockchain design built atop federated byzantine agreement, which has the ability to dynamically scale and be packaged as a Virtual Network Function (VNF) for the 5G Core.

## 1. Introduction

Unlike alternative distributed ledger structures, such as Block Lattice [1], Directed Acyclic Graph [2], and Distributed Hash Tables [3], which gain flexibility through modification or fragmentation of the underlying hashed-linked storage; blockchain allows little manipulation of its base structure outside of consensus model and block size. This rigid chain structure guarantees auditability, making it especially well-suited to policy-based operations demanding transparency and coordination among unmanaged network peers. For example, in events of natural disaster, or widespread infrastructure failure, having access to a trusted, secure, and decentralized data store, can be extended to allow infrastructure coordination, such as network slice allocation and other decentralised cyber-physical control, delivering neutral carrier emergency services to endpoints who would not otherwise be known subscribers.

Understanding that blockchain has the structural capability to allow for coordination among infrastructure peers, recent research has moved to focus on how to fit such coordination within existing and popular blockchain mechanics and, as a result, places incentive mechanics as core and requisite to operation. These range from shared infrastructure deployment of a virtual LoRaWAN network [4], resource management in neutral carrier [5], and 5G small cell deployment [6], as well as macro-level spectrum trading and management [7]. Beyond these, bespoke wireless network-specific forks of Ethereum have also been deployed to handle coordination of mesh infrastructure and last mile connectivity [8,9]. Underneath each of these is a limitation that is imposed by using a linear forward-hashed blockchain system that includes currency operations; they cannot be easily fragmented, since, by nature, currency transactions rely on the preceding balance recorded in perpetuity.

An inability to split up or retire ledger history has a secondary effect of reducing compatibility with the latest 5G and beyond network designs, which rely on virtual network functions with temporal/limited lifecycles and the ability to not only scale up, but also scale down. As network infrastructure is increasingly abstracted and replaced with software-defined platforms for 5G and beyond generations of deployment, use of blockchain allows for sharing and coordination in ways both known and unknown, and this shows further need for blockchain that is generalized and made widely compatible with cellular design. One way of doing this is returning blockchain to the function of ‘dumb’ storage and, in doing so, allow all cellular operations that store data, to make use of its decentralizing and immutable nature. On the path to generalizability in cellular deployment, it is important to recognize limitation in blockchain as a data structure, in that it is linear storage and, as such, does not scale in applications, where transactions have potential to be highly bursty, or expand exponentially, such as at the network edge. Recognizing this, and further time-bounds of edge operation, we target application of blockchain at the cellular core.

### Permissionless Blockchains Lack Lifecycle Control

In order to address concern of its monolithic structure and unbounded resource use, Ethereum has progressed through investigating a number of methods to scale down and make modular its monolithic blockchain, including horizontal data sharding [10], and state channels [11], as well as a full migration away from its original proof-of-work consensus, to a less compute intense implementation of proof-of-stake, named Capser, allowing for linear compute complexity ($O^{2}$) [12]. In each case, although more efficient, total storage remains unbounded, and without lifecycle, so the original difficulty to wholly package the system for temporal network function use remains. Further blockchain systems have focused on efforts to scale, but make no allowance for temporal use. Tendermint provides for scaling up and down by implementing federation in consensus that creates smaller clusters of consensus that overlap to guarantee a minimum byzantine fault tolerance [13]. These smaller clusters of consensus allow for controlled network segmentation and isolation, but lose the ability to deploy permissionless, as network topology and membership must be known to enforce its consensus cluster overlap for fault tolerance. The Stellar project is structurally similar to Tendermint, but it does not guarantee fault tolerance [14]. This federated model of consensus that removes fault tolerance but still guarantees safety and liveliness was named Federated Byzantine Agreement and first appeared with Stellar. Removing the guarantee of fault tolerance has the added benefit of allowing the consensus model to be used permissionless, but, because Stellar also includes a native currency, its ledger is monolithic and cannot be made temporal, so long as any participant carries a balance or need to transact on a previous history. In each of these systems, a work around for perpetual storage, and to assign a lifecycle terminus, is to use the systems in private deployment. In this model, a smaller group of participants can deploy ledgers for a single use and retire the ledger when that use is complete. However, in private deployment, these systems again lose any ability to function permissionless and, instead, behave in a manner similar to the permissioned and enterprise focused Hyperledger Sawtooth [15]. Table 1 provides an overview and comparison of these systems, as well as *Conte*, a new blockchain system presented in this research.

To the authors knowledge, we present the first permissionless blockchain which achieves the following properties:

- *Lifecycle Control:* Participants create single use chains that are immutable while being updated, and can be retired when no longer in use. By removing currency and contract functions, the remaining data storage function does not play a role in forward balance history, nor is it required for ledger security.
- *Network Function Compatibility:* As a data store, the blockchain is made agnostic to use case; combining this with lifecycle control allows for the system to be used for temporal 5G virtual network functions.

This research diverges from currency and contract focused research in two important ways; first, a deliberate focus is placed on blockchain use solely as secure, decentralized storage, rather than a mechanism of direct policy and incentive control; second, cellular design was given priority, with the goal of packaging blockchain to accommodate cellular operations rather than the inverse. This second goal, meaning to package blockchain as a standard virtual network function, one that can be scaled up, down, deploy, and to retire-allowing orchestration and lifecycle management, fitting 3GPP 5G Core [16], and Common API Framework [17] designs. To achieve this, a wholly new blockchain design is required. This research presents this design, which we name *Conte*.

The following research is split into six parts. The first provides an overview of the modular structure of the 5G cellular core, and presents areas where blockchain can be matured in order to improve its general compatibility by moving to a format as temporal general storage, rather than more prescriptive currency and contract designs. The second section details the consensus model used in Conte (Federated Byzantine Agreement), its safety, liveliness, and intentional omission of fault tolerance controls. Following these are details of our proposed Conte blockchain protocol, its block structure, protocol messages, and algorithmic complexity. The fourth section explains how Conte handles congestion and flow control, while a fifth section returns us to our initial cellular core context, to detail how Conte can be deployed as stand-alone temporal storage, or bundled as storage underpinning existing virtual network functions in real-world environments. Finally a conclusion is provided as a closing to the research, declaring potential improvements identified and planned future research directions.

## 2. Unbundling Blockchain for General Network Function Compatibility

Early blockchain research has focused largely on individual use cases and, as a by-product, makes a toy example of the wider cellular network dependencies. However, this framing does not fully acknowledge the heterogeneity of wireless networks whose hardware is modified and upgraded over time; for example, the coordination of Mobile Network Operators (MNO) and subscribing Mobile Virtual Network Operators (MVNO), where blockchain can ensure the verifiability of data, but each carrier operates its own diverging, and possibly competing services. A given MVNO may even utilize infrastructures across multiple MNO’s and, in this case, a level of compatibility, optionality, and generalizability of blockchain application would be desired.

When the 3GPP specification for 5G networks was released in 2018 [16], it explained its architecture as being comprised of many Network Service Functions (NSF) to support Network Function Virtualization (NFV) and Software Defined Networking (SDN) paradigms. It achieves this through modularity, separating hardware infrastructure into Control Plane (CP) and User Plane (UP) functions that are temporal, independently scalable, and loosely coupled to prevent structural dependencies when possible. Eighteen total service functions are identified within the “Architecture Reference Model” section of the 3GPP specification, and they are listed below:
Authentication Server Function (AUSF)Access and Mobility Management Function (AMF)Data Network (DN)Unstructured Data Storage Function (UDSF)Network Exposure Function (NEF)Network Repository Function (NRF)Network Slice Selection Function (NSSF)Policy Control Function (PCF)Session Management Function (SMF)Unified Data Management (UDM)Unified Data Repository (UDR)User Plane Function (UPF)Application Function (AF)User Equipment (UE)(Radio) Access Network ((R)AN)5G-Equipment Identity Register (5G-EIR)Security Edge Protection Proxy (SEPP)Network Data Analytics Function (NWDAF)

Today, there are two dominant paths of blockchain development. Either a bespoke chain can be created for the intended purpose, or a monolithic, single purpose chain may be deployed. Bitcoin contributed to the early work of Haber and Stornetta [18] in proving a system that could remain secure while being public [19]. As a digital currency, it was designed to be decentralized and permissionless; two traits not requisite in the original Haber and Stornetta digital notary use. To achieve this however, Bitcoin deploys resource intensive Proof-of-Work (POW) consensus that imposes throughput constraint and is difficult to deploy to resource constrained network environments, such as IoT. This conflict is manifested in examples, such as [20,21], which require the deployment of proxy devices that are able to run resource intensive POW calculations, or store the entirety of a public ledgers history, which is then referenced by appendage devices through an informal star topology. Taken in isolation, this full and light node separation can be understood as a symptom of the research relying on the POW variant of the Ethereum blockchain—but, in a macro perspective, represent a risk in network environments where the design and traits of a given blockchain evolve independently and potentially in conflict with wireless network design. An existing example evolution includes the introduction of Ethereum state channels, which impacts the auditability of data that would otherwise be stored in the main ledger [22].

Adopting a general use blockchain, such as Ethereum presents risk in that it lacks modularity of consensus, currency, contract, or other behaviors of operation. In Ethereum’s case, this means adopting behaviors to support a POW permissionless security model backed by currency functions which may be superfluous or even detrimental to the intended cellular network use. For example, if a universal record such as currency balance is not being mandated, it is then possible to form and retire chains for individual network operations as the shared data reaches the end of its useful life. Modularity of this type is not possible for the most popular blockchain systems, such as Ethereum.

### 2.1. Unbundling of Currency

The ability to use currency payment and reward in POW blockchains to incentivize behavior desired in network environments, such as resource sharing, was an early focus in cellular use. Taking a specific example, Maksymyuk et al. propose a spectrum sharing solution that identifies spectrum owners, infrastructure owners, ISP, and end users as independent participants in a dynamic market driven by the Nash equilibrium in game theory [23]. In the Maksymyuk model, end users make digital currency payments to infrastructure (base station) operators, who, in turn, pay for dynamic spectrum access to incumbents and regulators, while also paying for ISP backhaul services to carry traffic to the wider internet [23]. For specific controls relating to spectrum sharing operations, the research proposes a game theoretical scenario, where each operator has equal currency to use for spectrum access and it is incentivized not to overuse resources, as they would lose access once their balance reaches zero. The balance is only regained in this case, by supplying access to competing providers in a model that is designed for reaching Nash equilibrium of serving and receiving access. Although Nash equilibrium format is novel, it is, however, an example of a currency model that assumes a balance of infrastructure and customer that is difficult to guarantee in production networks. Another concern of the model is that it does not account for operations in congestion and peak demand scenarios where all of the participants have competing incentives to consume access, risk service disruption, or total service outage.

To best fit existing mechanics of network environment, blockchain must be evolved to function under competing operational incentives, such as resource management, network investment levels, and demand growth, which may be uneven among equivalent providers. One way of servicing this structure is through the sharing of context and data, such as tower location and channel occupation-which are required for functional operation of everyone—decoupling any awareness of economic model from the chain. Conte fully removes currency, for generalized network use.

### 2.2. Unbundling of Contract

Blockchain is a rigidly time ordered structure by nature of its linear forward hashing. The general speed of code execution tied to the contribution of blocks will be inversely proportional and dependent on block consensus time. This means that a blockchain deployment seeing an increase in block contributions will also see a corresponding decrease in how quickly those blocks and corresponding information can be processed; all else remaining unchanged. In systems, such as Ethereum, where end-to-end operations occur within its own virtual machine-behavior controls again fall back to currency incentive, where impacts of block additions can be partially controlled by charging a digital currency fee proportional with delay being imputed on the system [24].

**Figure 1:**
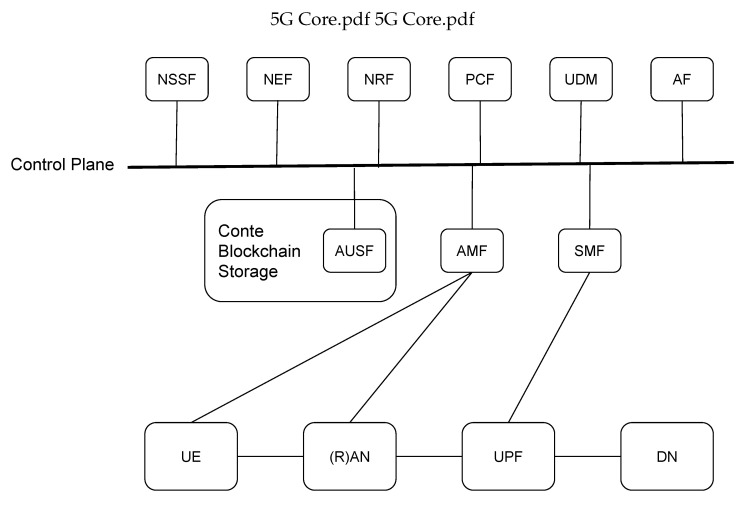
A logical representation of 5G network functions, with a single function (AUSF) being decentralized by using Conte blockchain storage.

Modern networks are built using an unbounded variety of hardware configurations and radio resource management algorithms. Within networks of an identical generation, configuration for antenna geometry, sectoring, deployment density, backhaul capacity, and algorithms deployed to maintain quality and coverage can differ and conflict among networks and be further modified in time. Contractual code execution assumes a level of heterogeneity and coordination that does not fit with existing or expected future network design. Contracts cannot easily account for all possible transitions in heterogeneous networks or multiple operators. The latency of contract checks and block propagation cannot be completed at sub-millisecond scale, as required for time varied channel conditions/controls. It is possible to modify the algorithms for achieving consensus to get around these limitations. Blockchain systems can be manipulated to process higher transaction volumes, or also control resource usage by allowing for nodes to keep full (full-node) or partial (light-node) states [25]. Deploying such modification however, shows consensus latency in blockchain under best case scenarios, are reduced to one second [26]. This present scalability limitation reveals blockchain structure as largely incompatible with operations at μ-second scale at the network edge, such as real-time radio resource control and dynamic accesses not set on a semi-permanent basis. This again reveals a general benefit of limiting blockchain deployment to the decentralization of data. In part to mitigate known limitations of contract execution and corresponding cyber-physical control bound by block delay, Conte fully removes the function of contracts for generalized network use. In doing so, network operators can still share data in an immutable, decentralized record, while also independently updating and swapping out systems of cyber-physical control over time. The integration of Conte within a 5G system assumes a Cloud-Native 5G Core, whose composing functions/services (e.g., AUSF, NEF, etc.) are exposed via well-defined APIs (e.g., CAPIF, ETSI NFV IFA 013, etc.) under the Mobile Network Operator’s (MNO) policies. Conte operates as the Unstructured Data Service Function, making it not specific to any single network function, but rather it is agnostic storage that can be accessed and used by any network function, as defined by the previously mentioned 3GPP 5G Architecture Reference Model. This allows a network to decentralize storage and accounting for all or just a smaller subset of network functions. Figure 1 shows a logical example, where only a single network function (AUSF) uses the Conte blockchain for its storage, while all other functions retain an unmodified design.

## 3. Federated Byzantine Agreement Consensus

A consensus model must be deployed that functions in this mode of operation to make a blockchain that is temporal. The following section details how the Conte blockchain maintains safety, while removing currency and contract mechanics from its design.

When compared to permissionless consensus models, such as Bitcoins Proof-of-Work (POW), classic Byzantine Fault Tolerant (BFT) algorithms have been favored for permissioned or consortia deployments due to its lower resource consumption achieved in exchange for a reduced and adjustable fault tolerance, commonly set as low as 20% for environments consisting of known peers [13]. However, BFT models, in turn, carry risk in lacking the standardization and interworking required of heterogeneous network deployment. A more recent approach extended for this research is the FBA model, which balances the permissionless decentralization of POW, with the lowered resource use of BFT consensus.

Conte handles consensus while using a modified implementation of the Federated Byzantine Agreements (FBA) structure first introduced with the Stellar Consensus Protocol [14]. FBA functions by dividing networks into smaller clusters of interlinked consensus, aptly named ‘slices’. Partitioning the network into slices in this manner allows for deploying BFT agreements at internet scale, while making the trade-off of slower consensus speed. Functionally, these slices behave in a manner similar to network subnetting, eliminating traffic storms formed during broadcast consensus in existing Byzantine Fault Tolerant (BFT) algorithms and, at a macro level, allows for consensus to mirror the unbounded peer-wise model of backbone internet, with nodes spanning consensus slices, functioning as gateway.

Modern blockchain consensus algorithms are characterized on the three matrices of safety, liveliness, and fault-tolerance. The FLP Impossibility Theorem states that any asynchronous consensus mechanism can only guarantee and choose two among the three [27]. Extending from this, FBA diverges from Classic BFT consensus in being asynchronous and, consequently, foregoes guarantees of fault tolerance. An example BFT consensus algorithm guaranteeing 25% fault tolerance uses an n ≥ 3***f*** + 1 security model, with total nodes *N*, faulty nodes *f*∈*N*, and n = { x∈N ∣ x∉*f* }. For such a guarantee to function, classic BFT algorithms must, at minimum, operate in partial-synchrony, often using a global stabilization time (GST) to end voting, and with a known registry of nodes *N* in order to reliably identify *f* nodes at a given time *T* [28,29].

Through choosing safety and liveness over fault tolerance in its core algorithm, Conte does not need to restrict participation or incentivize behavior among unmanaged nodes in order to reach agreement. In this manner, it functions in a manner mirroring internet backbone peering; where connectivity is piecemeal, extending unbounded in all directions, and changing in time-based on trust relationships not managed by the blockchain itself. In this structure, Conte offers an ideal starting point, allowing for blockchain connectivity to be locally managed under existing infrastructure paradigms (as temporal virtualized network functions), while safely settling and distributing finalized blocks among unmanaged and heterogeneous networks. For this research, we define safety and liveliness as:

- Safety: nodes operating a Conte blockchain enjoy safety if node outputs are consistent, with no two nodes committing a conflicting values for the same block.
- Liveness: nodes operating a Conte blockchain enjoy liveliness if they are able to reach consensus on new blocks without the participation of failed or malicious nodes.

### Replacing Fault Tolerance with Network Quorum

Consensus in FBA’s operates on a structure known as a quorum slice. A quorum slice is a grouping of network peers whose pairwise peering is symmetric. Transposed to wireless network context, a quorum slice could consist of all tier-1 mobile network operators (MNO) of a region, who all peer with each other. Within a quorum slice, block additions may be considered final, after a threshold amount of peers confirm the block. However, this functionality on its own does not consist a federation. To form a federation, quorum slices are intended to intersect, such that nodes operating in multiple quorum slices function as relay, extending consensus to the wider network of intersecting slices. In the 5G core network context, this would occur when some portion of regional MNO’s within a quorum slice, also peer with MNO’s or another region, or internationally. Federated Byzantine Agreement Systems (FBAS) and Quorum are formally defined as [14]:

- Federated Byzantine Agreement Systems: a federated Byzantine agreement system, or FBAS, is a pair 〈V,Q〉 comprising a set of nodes *V* and a quorum function $Q:V\Rightarrow 2^{2^{V}}\setminus \left\{\emptyset \right\}$ specifying one or more quorum slices for each node, where a node belongs to all of its own quorum slices—i.e., ∀v∈V,∀q∈Q(v),v∈q. (Note that $2^{x}$ denotes a powerset of *X*.)
- Quorum: a set of nodes U⊆V in FBAS 〈V,Q〉 is a quorum iff U≠∅ and *U* contains a slice for each member—i.e., ∀v∈U,∃q∈Q(v), such that q⊆U.

**Figure 2:**
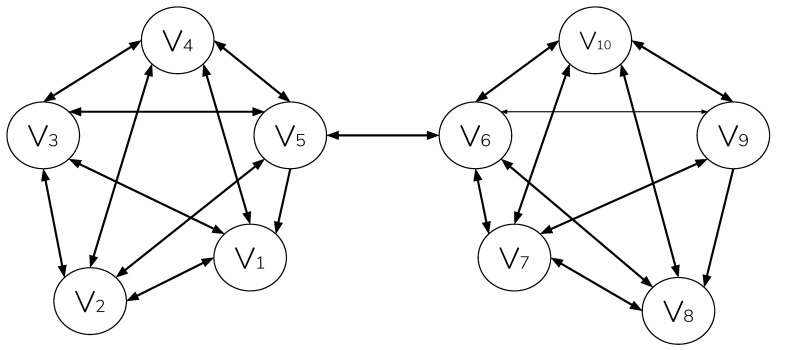
Two quorum slices, intersecting at nodes $\left\{v_{5},v_{6}\right\}$.

A quorum is a set of nodes that sets the threshold or reaching agreement, and it may be larger than a single quorum slice. Consider Figure 2, which shows two clusters of nodes, each participating in a single quorum slice with symmetric pair-wise connection. Assuming that 100% confirmation is required, node $v_{5}$ can reach agreement with confirmations from peers $\left\{v_{1},v_{2},v_{3},v_{4},v_{6}\right\}$; however, since node $v_{6}$ has additional peers $\left\{v_{7},v_{8},v_{9},v_{10}\right\}$, they must also agree to the update before it is accepted; therefore, $v_{4}$ must agree to an update from $v_{8}$, etc.

**Figure 3:**
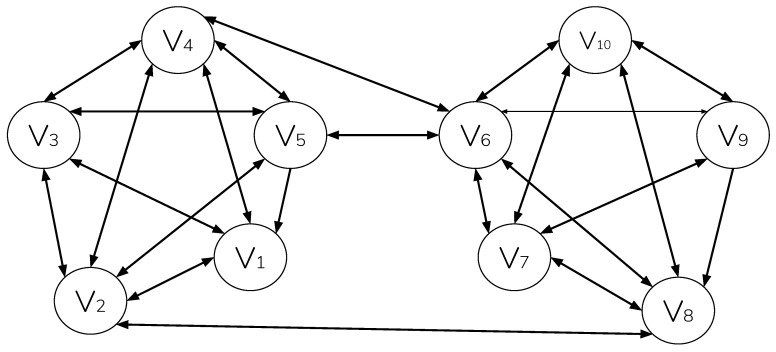
Two quorum slices intersecting at nodes $\left\{v_{2},v_{4},v_{5},v_{6},v_{8}\right\}$.

Figure 2 represents a worse case scenario of federated agreement. In this example, the pairwise relationship of $v_{5}$ and $v_{6}$ represent a single point of failure in reaching consensus. Systems, such as Ripple [30], compensate for this by enforcing, at all times, a minimum connectivity between federated nodes ≥ its maximum fault tolerance (and in turn, making it Byzantine Fault Tolerant). However, doing this again imposes the centralizing requirement of recording and enforcing connectivity among known participants-precluding unmanaged permissionless operation. FBA, as deployed in Conte, instead makes an alternate scenario possible, in which consensus resilience increases as additional unmanaged pair-wise relationships are formed elsewhere in the network-in a manner mirroring that of global internet (Figure 3).

## 4. The Conte Blockchain Protocol

Conte operates permissionless, without an explicit membership or validator set; each block, however, is signed using the public key of the submitting node and is by design, not anonymous. Each block update is assigned an incrementing index number, such that only one block can be valid at a given index position, with each node able to independently confirm block sequencing against its local set (its local blockchain). Block submissions are then forward propagated until reaching a graph edge, where edge nodes begin a ripple effect through the back-propagation of an acknowledgment for a given block vote [30]. Blocks that are settled in consensus are then added to local blockchains using SHA-256 encryption. Because Conte is intended to operate with an unknown number of peers, there are no leader election processes, or transaction batching as done in Byzantine Fault Tolerant blockchain systems, such as Facebook’s Libra [29]. Rather, any participating node can submit a block at any time, relying on congestion control measures borrowed from medium access controls in IEEE 802.11 networks (carrier sensing multiple access with collision detection (CSMA/CD)). Exponential back-off timers [31] within Conte allow peers to send a negative acknowledgment, triggering a cool down period for a proposing node if receiving blocks out of order, or with conflicting index values to those received from disjoint peers; the details of this mechanism are provided later in the paper. Conte further combines these network behaviors with novel federated byzantine agreement consensus, which establishes finality without traditional fault-tolerance, to allow its temporal network function deployment, while also not sacrificing consensus safety.

### 4.1. Block Structure and Storage

Blocks within Conte are composed of three parts; the block header, a list of transactions, and the previous block hash. Rather than a bespoke programming language and contract syntax, Conte flattens and standardizes possible operations to aid in interworking between networks, in a similar manner to IP packet structure. Each transaction is atomic and contains all of the information required for processing operations of the specific network function for which it is deployed, while also adhering to a single global format containing the sections below.

- Contract ID: a globally unique integer value, serving as the identity of an Federated Byzantine Agreement (FBA).
- Contract Name: a non-unique string value, serving as a human readable name for a given FBA.
- Message Signature: the cryptographic signature, or public key of the network node proposing a transaction.
- Function: a rigidly defined struct value-defined as standard for each network function. An example struct being: [NF Name]; [Operation]. In the AUSF use case, this would designate: [AUSF]; [authorize], [AUSF]; [revoke], or others standard operations of the chains’ designated network function.
- Message Body: an array value containing the core transaction data. In the example AUSF use case, this is the 5G Globally Unique Temporary Identifier (the 5G subscriber ID).
- Index Number: an incrementing integer value, designating a records position in the hashed chain.

Conte is structured to allow a node to participate in multiple independent chains simultaneously. Rather than a monolith chain that grows in perpetuity, Conte intends new chains to be created, run in parallel, and retired after serving an intended purpose. This managed lifecycle can be months, years, or an indeterminate amount of time; partner networks may, for example, share subscriber data to grant access in cases of natural disaster. Because Conte requires full agreement to settle consensus, meaning that all peers of a given node must provide block confirmation before a block is considered to be final; there is no risk of fork and no increase of security through increasing the ledger length in perpetuity. This paradigm facilitates the negotiation of software upgrades among smaller subsets of peers for individual chains, and also with consideration to the expectation that network data is real-time data, whose value trends towards zero in time.

**Figure 4:**
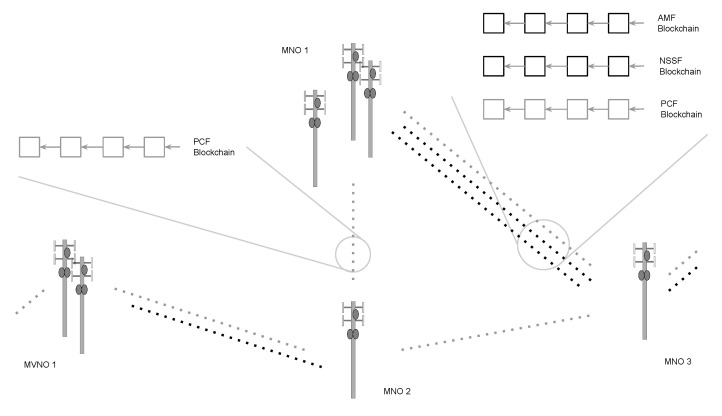
Example of multiple network function-specific blockchains running across operators.

Because the Conte blockchain stores living network data, it is important to allow a mechanism to prune or optimize storage. This is done in two ways; during an initial sync or in ongoing pruning. Returning to our example scenario of authorization, let us assume a network requirement where devices must be reauthorized every 90 days through a transaction renewing its permissions. Assuming that each network has an external record retention policy and mechanisms of network logging, this effectively places a 90-day expiry on the utility of transactions in the chain. In a scenario such as this, a node requesting to sync can do so by requesting all transactions from *N* date, rather than an entire chain growing in perpetuity. Because the chain length is not a mechanism of security, as in Ethereum, or assisting in reaching finality as in IOTA, the chain can be partially synced in this manner without risking safety of ongoing consensus, as defined by the intended use of the single chain, or microchain (Figure 4).

### 4.2. Transaction and Protocol Messages

Joining consensus on a Conte blockchain occurs by configuring a peer and mutually validating identity through exchange of public keys. In addition to public key exchange, a peering request arrives with either a genesis block, containing a randomly generated contract ID, or a request to sync, containing the contract ID of an existing chain that is the target of synchronization. It is assumed that 5G core networks are connected pair-wise, rather than fully peer-to-peer. Structuring consensus in this way allows it to function when networks are operating peer-to-peer, but also when sensitive infrastructure is siloed behind firewalls and strict route controls.

Nodes may also proxy or pass block proposals while using NEF operations, in compliance with 3GPP 5G design. Conte exposes secure RESTful APIs for protocol messaging between nodes, including GET and POST operations. Protocol messages include: peer, sync, propose, acknowledge, negative acknowledge, commit, and prune.

**Figure 5:**
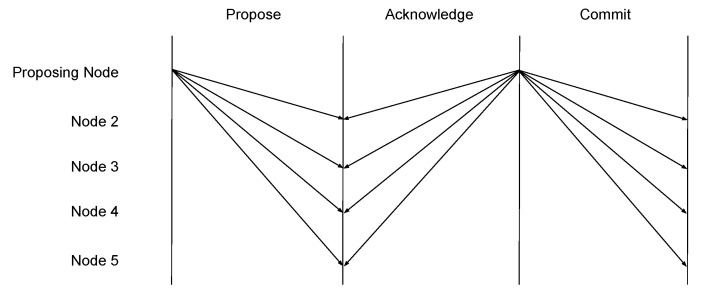
Conte Single Round Block Commit.

- Peer: initialize connection to new contract peer.
- Sync: after initial connection, or during conflicting commits, a node can request to sync transactions. This sync includes a check to confirm a known peer with the longest change, and a verification of new blocks by re-hashing them using the SHA-256 algorithm.
- Propose: issue a new transaction. A new block can be proposed by any member of the network. The block must be signed with a cryptographic key to validate identity. Each block is sent as unicast to all peers of a given node, which forwards the proposal, until reaching nodes at the graph edge. For bandwidth efficiency, a node may delay block proposal, until it has multiple transactions to submit-in which case, these may be bundled into a single block. A node is considered a graph edge after receiving the same proposed block from all its known peers.
- Acknowledge: provides confirmation of acceptance of new transaction blocks. Once a proposed block is received, its header, body, and signature data is validated and an acknowledgment is returned. A node must receive an acknowledgment for each forwarded block proposal, before propagating back its own aggregate acknowledgment. Nodes receiving invalid blocks, competing blocks with the same index value, or out of sequence blocks send a negative acknowledgment, aborting consensus.
- Negative Acknowledge: deny confirmation of blocks in case of conflicting block data, such as index position, aggregate hash, or message signature.
- Commit: a final notification that a block is committed locally by a proposing node, signaling that remaining nodes handling consensus as clear to add the block into their respective local chains. Conte ensures that no two nodes store different blocks with the same index value, by aborting consensus when encountering conflicting data or error. In states where acknowledgment is received from all peers, the block is committed to the local chain and a final commit message is issued to peers (Figure 5), who commit the block in their local chain and propagate the commit forward toward edge nodes.
- Prune: nodes that fail to reach consensus may initiate a request to prune from peer lists, any peer which has failed to respond to three consecutive proposals. Prune requests forward propagate in a manner that is similar to a propose message, with the full network reaching consensus to prune the peer. A negative acknowledgment may be sent if the target peer is responsive elsewhere in the network.

Because Conte requires full confirmation in order to reach finality in a manner that is similar to TCP error correction, a given message is repeated if an expected response is not received within a given timeout, until a pruning state is triggered. Algorithm 1 shows simplified pseudo-code of Conte message functions.


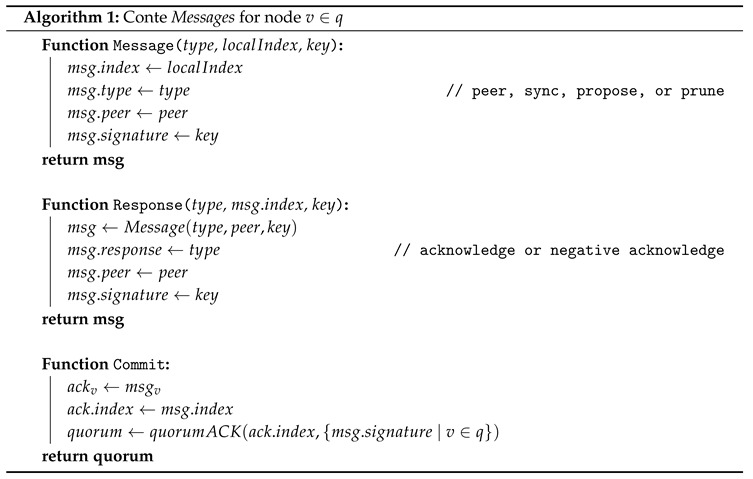


## 5. Handling Transmission Contention

As an asynchronous system without a static resource allocation, additional controls are needed in order to handle contention in transmissions. In addition to time-to-live limits on transactions, Conte handles conflict in the network using a binary exponential back-off mechanism, mirroring that of CSMA/CD in WiFi networks. The channel sensing in this case is the monitoring of the directly connected peered network interfaces for the transaction traffic of peer nodes. Under the CSMA/CD model [31], a transmitting node must wait some minimum sensing period in order to confirm the transmission medium is idle. In practice, if the transmission medium is not idle, then the node selects a random back-off duration, in seconds (contention window), and counts down. This back-off is chosen uniformly in the range $\left[0,2^{i}W_{0}-1\right]$, where *i* is the number of times a node has attempted to issue the transaction (back-off stage), initialized at 0, and $W_{0}$ is the minimum sensing period [32]. If a node receives subsequent transactions during the sensing period, it pauses its countdown, and continues decrementing once the transmission medium is clear. After transmitting successfully, *i* is reset to 0. A maximum *m* number of retransmissions attempts *i* is also set, to apply a bound for maintaining liveliness. Again, matching the behavior of CSMA/CD, a node makes two attempts at the maximum back-off stage, before considering the block a failed transmission. This mechanism of congestion control is chosen, as it represents a worst case scenario in which only directly connected peers are known-with no visibility beyond, as opposed to Reno, Tahoe, and other congestion control algorithms available directly in TCP, which require maintaining a route table that eventually extends to includes all graph hosts. A summary pseudocode of Conte consensus, inclusive of back-off timing, is provided in Algorithm 2.

The CSMA/CD model provides four contention probabilities that can be represented as a two-dimensional Markov chain with one step transition probabilities, as explained in [32] with possible states represented where *t* in our case is a block retransmission attempt, and *s* is the sensing period. These Markov transition probabilities are represented as (1), where $P_{w}$ is the contention probability of the transmission medium, $W_{0}$ is the minimum sensing period length, $W_{i}=2^{i}W_{0}$ is the sensing period length at a given block attempt *i*, and i=m at the maximum retransmission attempt:

(1) $$\left\{\begin{array}{ccc} P\{t,s|t,s+1\}=1, & s\in \left(0,W_{i}-2\right) & t\in (0,m+1)\\ P\left\{0,s|t,0\right\}=\frac{1-P_{w}}{W_{0}}, & s\in \left(0,W_{0}-1\right) & t\in (0,m+1)\\ P\left\{t,s|t-1,0\right\}=\frac{P_{w}}{W_{j}}, & s\in \left(0,W_{i}-1\right) & t\in (1,m+1)\\ P\left\{0,s|m+1,0\right\}=\frac{p_{w}}{W_{0}}, & s\in \left(0,W_{m}-1\right) \end{array}\right.$$

In descending order, these probabilities (1) are the transition probability of going from idle to successful transmission; the second representing the transition probability after successful transmission of having a subsequent successful transmission; the third represents the transition probability after an unsuccessful transmissions, in which the contention window $W_{0}$ is doubled, as defined by $\left[0,2^{i}W_{0}-1\right]$; the last equation represents the transition probability after a fully failed transmission in which the contention window resets to 0. Letting an expired timer (or closed contention window) be represented as $b_{t,0}$, accounting for the distribution of Markov transition probabilities, the probability of a node sending a block in any 1 s time period $\tau_{w}$ is represented as (2):

(2) $$\begin{array}{c} \tau_{w}=\sum_{t=0}^{m+1}b_{t,0}=\frac{2}{W_{0}\left(\frac{\left(1-{\left(2P_{w}\right)}^{m+1}\right)\left(1-P_{w}\right)+2^{m}\left(P_{w}^{m+1}-P_{w}^{m+2}\right)\left(1-2P_{w}\right)}{\left(1-2P_{w}\right)\left(1-P_{w}^{m+2}\right)}\right)+1} \end{array}$$

A given node can only listen to the transmission medium of its connecting peers within a single quorum slice and, consequently, nodes on disjoint slices are occluded. To compensate for this, it is assumed that the binary exponential back-off is triggered either by listening directly on the transmission medium, or by a known peer sending a negative acknowledgment on a transaction, as done in cases when it has already received a superseding transaction time stamp or index position from elsewhere in the network.


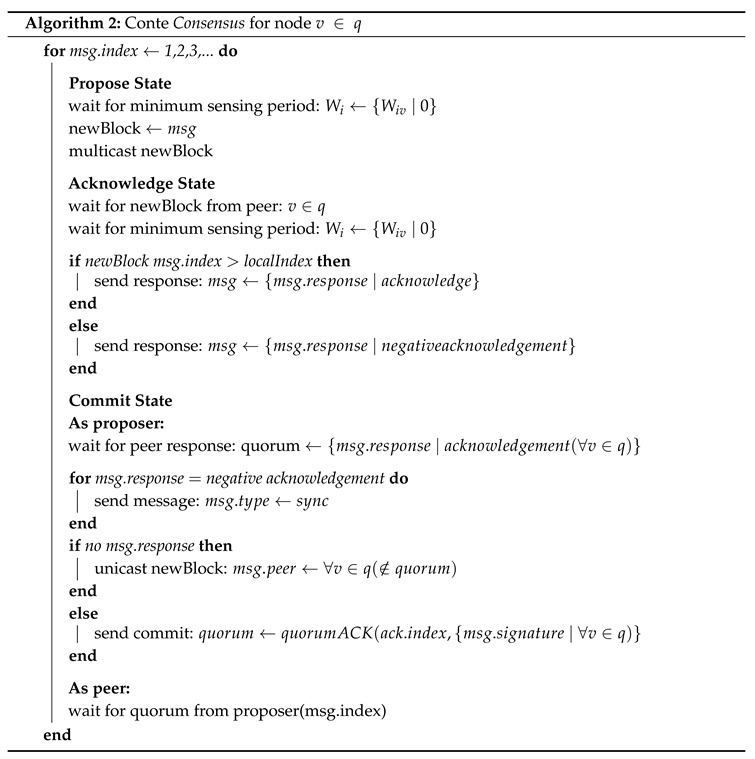


## 6. Performance and Scalability

Because Conte does not deploy topology constraint, it is valid to represent its network scalability as a model of congestion control, where the performance bounds of the total system are held by link propagation and block contribution rate. Two simulations were implemented in Python in order to model the scalability of Conte CSMA/CD congestion control. In the first simulation, peer nodes were placed equidistant at 1500 km apart, roughly the distance between the cities of Barcelona and Berlin. For the second simulation, peer nodes are set an order of magnitude further at 15,000 km, representing the equivalent distance between Los Angeles and Singapore. These two distances allow for evaluating Conte under both regional and global network delay.

All other parameters were set identical, with link speed of all nodes at 1 Gbps and block size at 1500 bits. Because of the randomness introduced through retransmissions using a binary exponential back-off, each simulation was run ten times, with the results taken as the average. The simulations were run in two sets, one with node sizes ranging between two and 10 peers, and a second with node sizes that range between 10 and 50 peers. It is important to note that node sizes do not represent the absolute number of possible network participants, rather the maximum number of peer hops between the network graphs furthest edges, to present a worst case. As a system designed for policy orchestration at the cellular core, the first simulation set with a node maximum value of 10 is representative of cloud native deployment, where regional data center and points-of-presence (POP) locations potentially house carrier cores. The second set with node sizes reaching 50 represents an outlier scenario of possible network loops or misconfiguration. Conte simulation code is available online and it has been open-sourced [33].

**Figure 6:**
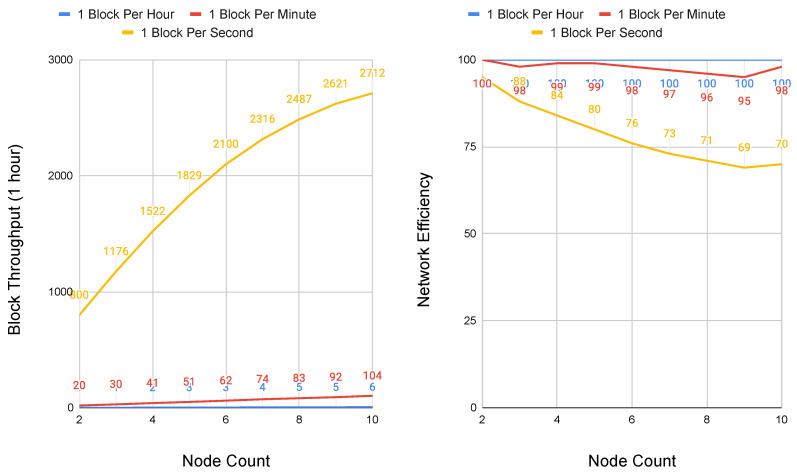
Performance and scalability of Conte at 1500 km node distances and maximum hops to graph edge of 10. Block Throughput (**left**) and Network Efficiency (**right**).

The first simulation that is depicted in Figure 6 shows total block throughput and network efficiency for three rates of block contribution: one block per hour, one block per minute, and one block per second. Simulating block contribution at orders of magnitude is done to reflect a wide range of update frequencies possible across 5G core network functions. At 1500 km, block throughput scales up to handle network updates at rates as fast as 1 block per second without significant degradation. Network efficiency, measured as the percent of packets transmitted successfully as compared to total packets, reduces as low as 69% at this peak load. At 1500 km, both block throughput and network efficiency scale linearly at rates below one block per second.

**Figure 7:**
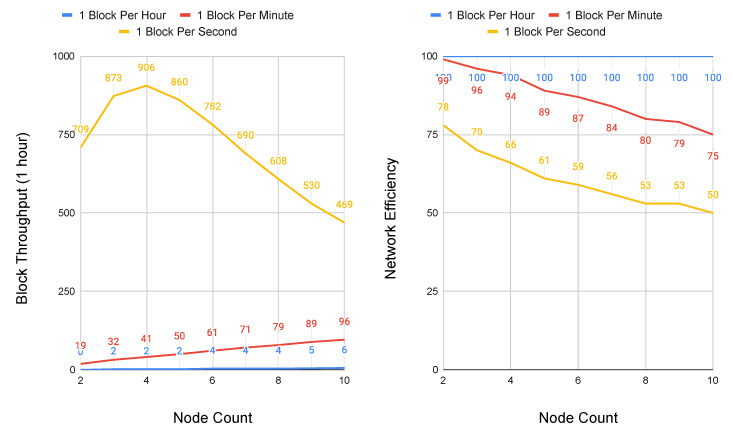
Performance and scalability of Conte at 15,000 km node distances and maximum hops to graph edge of 10. Block Throughput (**left**) and Network Efficiency (**right**).

At 15,000 km, Figure 7 shows the performance roll off as a result of the additional network delay. In this simulation, as delay increases an order of magnitude, block contribution capacity drops correspondingly, with the network only able to scale to 1 block per minute without significant degradation. Network efficiency above this rate falls as low as 50%, with the additional overhead of retransmissions causing sustained reductions in block throughput beyond four graph hops at the one block per second rate. The one block per second contributed rate also shows how the system degrades under abnormally large network delays.

**Figure 8:**
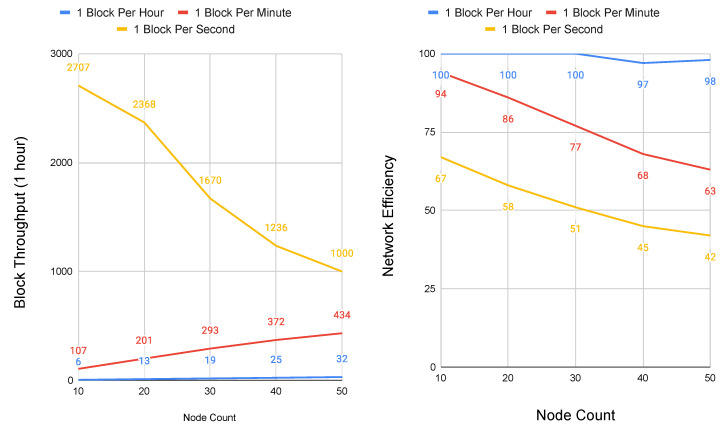
Performance and scalability of Conte at 1500 km node distances and maximum hops to graph edge of 50. Block Throughput (**left**) and Network Efficiency (**right**).

Increasing node sizes to 50 at 1500 km (Figure 8) shows marginal impact at block rates of one per hour, while network efficiency begins to fall sooner, seeing reductions at the 1 block per minute transmission rate that was previously little impacted at smaller node sizes. It is important to note that overall throughput does continue to improve, and remains above the peak of the initial simulation set which capped node sizes at 10. With one block per second rates at 1500 km, we see the system become overwhelmed with block throughput dropping sharply, having a throughput rate at 50 nodes that is below that of a system having only three in the first simulation.

**Figure 9:**
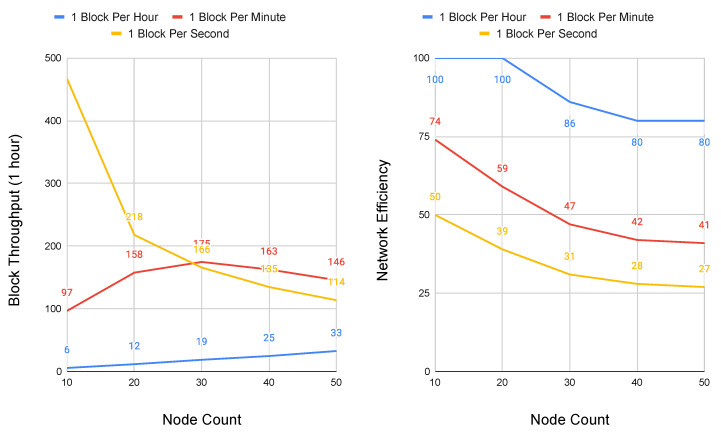
Performance and scalability of Conte at 15,000 km node distances and maximum hops to graph edge of 50. Block Throughput (**left**) and Network Efficiency (**right**).

Simulation results at 15,000 km (Figure 9) exhibit similar behavior, and they continue a downward trend already present at smaller node sizes. An interesting behavior we can see in aggregate is that network efficiency measures do not drop to levels suggested by the raw block throughput numbers. This can be explained by the CSMA/CD algorithm implementation being assigned a maximum retransmission attempt value of 10. Network efficiency as reflected here, does not consider a packet dropped, until it has already attempted transmission 10 times. Adjusting this maximum retransmission attempts also has potential to impact performance, but this is outside the scope of this writing.

## 7. Deploying Conte as a Temporal Network Function

Conte is intended to be packaged as standalone temporal storage, or inside of other Virtual Network Functions (VNF) within an operators’ Network Functions Virtualization Infrastructure (NFVI). Delivering a Conte node to the 5G core network is relatively straightforward thanks to virtualization technology. 5G adopts virtualization approaches commonplace in cloud data centers to realize virtual network functions (VNF), as opposed to relying on traditional network functions with a tight coupling between software and hardware, as mentioned previously. VNFs, or more generally, a Virtual Function (VF) can be thought of as a block of functionality running within a virtualization container (e.g., Virtual Machine, containers), which can then be connected together via Virtual Links (VL) and Software Defined Networks (SDN) to provide a service (e.g., 5G Core). In 5G, the way VF is described, deployed, managed, and destroyed has been defined by the ETSI NFV group [34]. Furthermore, resource and service isolation in multi-service multi-tenant environments is achieved via the concept of 3GPP Network Slices, which effectively treats a collection of VFs as a single administrative entity, allowing for administrators to scale VFs individually, destroy or create multi-VF services [35].

**Figure 10:**
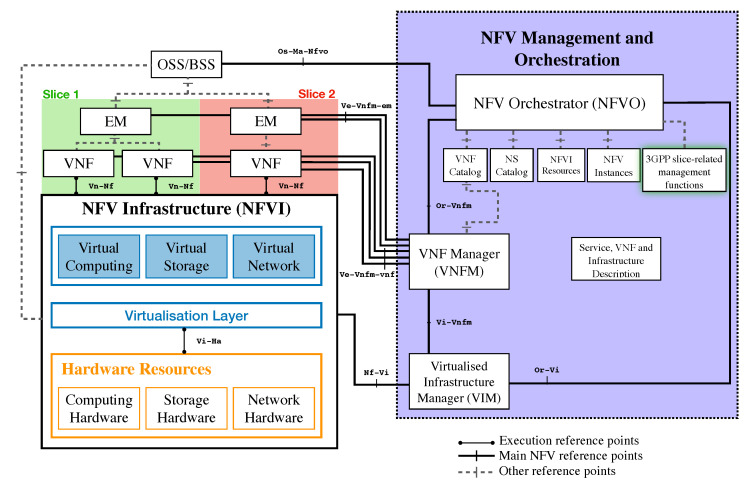
ETSI NFV MANO Architecture: highlighting slices’ reference points and manager in an integrated NFV MANO model [36].

Conte can be deployed as a VNF in an operators’ Network Functions Virtualization Infrastructure (NFVI). An example architecture of such deployment is provided in Figure 10. In Figure 10, the standard NFV Management and Orchestration (MANO) architecture is displayed, including reference points that enable communication among its components [36]. Moreover, two example slices (labeled Slice 1, and Slice 2) are displayed to describe how VNF’s share an underlying NFVI, which also allows communication among slices via Virtual Networks.

From the architecture proposed in Figure 10, Conte orchestration on operator’s NFVIs can be realised as a separate slice (e.g., to isolate life cycle management operations), or as an additional VNF within 5G Core Network slice (or equivalent).

### 7.1. Evolving Alongside a Cloud Native 5G Core

In order to achieve the advertised dynamicity and reconfigurability of the 5G core (e.g., placing UPF at the network edge), its implementation is expected to evolve towards stateless micro services [37]. Such micro services can be considered equivalent to VF, albeit often referred to as Container Network Functions (CNF), because they are implemented within lightweight virtualization containers (e.g., Docker containers). Such move towards micro services would allow a new set of functionalities (e.g., rolling updates, roll-back), and capabilities (e.g., placement of functions at resource-constrained devices at the edge, admit automation), while increasing performance when compared to VNF due to container’s reduced virtualization overhead.

Conte may be deployed as cloud-native application (i.e., a collection of micro services inside Docker containers) on top of a Platform as a Service (PaaS) provided by the operator, e.g., following ETSI NFV IFA 029 recommendations [38]. Such a PaaS may hold a Container Infrastructure Service (CIS) instance configured with a NFV MANO-compatible Container Orchestration Engine (COE, e.g., Kubernetes, OpenShift). Such a CIS will then support a cloud-native Conte, as well as expose network resources to reach the operator’s 5G core.

### 7.2. Conte’s Carrier Security Model

Conte is permissionless, but not trustless. A limitation of the proposed Conte architecture is that it does not enforce any specific security model for the chain itself; an approach differing from blockchains designed to handle byzantine faults (BFT/PBFT) or incent network behavior through currency reward (POW). This model allows for decoupling currency and code execution from the underlying immutable storage and decentralized consensus of the blockchain, at the expense of security in isolation. This modified format is what allows Conte to achieve a 3GPP 5G Architecture compliant design.

By not directly enforcing security within the chain, Conte inherits SDN rules, and other security measures that are specified in 3GPP’s Common API Framework (CAPIF) [17] to guarantee secure and interoperable access to 5G Core functions (e.g., NEF)-both internally and across carriers. Beyond 3GPP-defined CAPIF controls, it is still possible for a peer carrier to broadcast malicious or misconfigured updates, making Conte vulnerable to update poisoning in a manner similar to BGP route poisoning. For this reason, it is assumed that a carrier operates Conte blockchains only with trusted peers. As peering expands, Conte remains permissionless in membership, but it does not support a trustless model.

## 8. Discussion

Recently, blockchain and other distributed ledger systems have received increased attention as a means of augmenting cellular networks. Using existing systems, such as Ethereum and Bitcoin, however, requires accepting both a monolithic, never-ending ledger structure, as well as currency and contract models that are not a native fit in existing cellular design. This paper introduces and details a new blockchain protocol, named Conte, designed with a temporal structure, more suitable for expiring data and as storage backing native network function with a known lifecycle terminus, fitting within 3GPP and ETSI defined 5G dynamic function design. The Conte design that is presented in this research is permissionless, decentralized, internet scalable, and structured to handle contention, remaining immutable during its deployment lifecycle. To the authors knowledge, Conte is the first blockchain system to be both permissionless and allow lifecycle control. The simulation results show the system scales in regional deployment with block contributions as frequent as one block per second, while global deployment supports block contribution at 1 per minute. As embedded carrier infrastructure, the Conte design as proposed does not support trustless operation. Further investigation in support of a trustless model has been identified for future research.

## Figures and Tables

**Table 1:** Comparison of selected blockchain distributed ledger systems.

Ledger	Consensus	Compute Complexity	Model	Currency	Temporal
Ethereum (Casper) [12]	Proof of Stake	O(n)	Permissionless	Yes	No
Tendermint [13]	BFT	O(n)	Permissioned	Yes	No
Hyperledger Sawtooth [15]	PBFT	O($n^{2}$)	Permissioned	No	Yes
Stellar [14]	FBA	O($n^{2}$)	Permissionless	Yes	No
Conte	FBA	O(n)	Permissionless	No	Yes

## Abbreviations

The following abbreviations are used in this manuscript:

3GPP	3rd Generation Partnership Project
5G-EIR	5G-Equipment Identity Register
AF	Application Function
AMF	Access and Mobility Management Function
API	Application Programming Interface
AUSF	Authentication Server Function
BFT	Byzantine Fault Tolerant
BGP	Border Gateway Protocol
CAPIF	Common Application Programming Interface Function
CIF	Container Infrastructure Service
COE	Container Orchestration Engine
CP	Control Plane
CNF	Container Network Function
CSMA/CD	Carrier Sensing Multiple Access Collision Detection
DN	Data Network
ETSI	European Telecommunications Standards Institute
FBA	Federated Byzantine Agreement
FBAS	Federated Byzantine Agreement System
FLP	Fischer, Lynch, and Paterson
GST	Global Synchronization Time
IFA	Interfaces and Architecture
ISP	Internet Service Provider
LoRaWAN	Long Range Wide-Area Network
MANO	Management and Orchestration
MNO	Mobile Network Operator
MVNO	Mobile Virtual Network Operator
NEF	Network Exposure Function
NF	Network Function
NFVI	Network Function Virtualization Infrastructure
NFV	Network Function Virtualization
NRF	Network Repository Function
NSF	Network Service Function
NSSF	Network Slice Selection Function
NWDAF	Network Data Analytics Function
PaaS	Platform as a Service
PCF	Policy Control Function
POP	Point of Presence
POW	Proof-of-Work
RAN	Radio Access Network
REST	Representational State Transfer
SDN	Software Defined Networking
SEPP	Security Edge Protection Proxy
SMF	Session Management Function
TCP	Transmission Control Protocol
UDM	Unified Data Management
UDR	Unified Data Repository
UDSF	Unstructured Data Storage Function
UE	User Equipment
UP	User Plane
UPF	User Plane Function
VF	Virtual Function
VL	Virtual Links
VNF	Virtual Network Function
